# *Toll-like receptor* genetic variations in bone marrow transplantation

**DOI:** 10.18632/oncotarget.17315

**Published:** 2017-04-21

**Authors:** Kaori Uchino, Shohei Mizuno, Aiko Sato-Otsubo, Yasuhito Nannya, Motonori Mizutani, Tomohiro Horio, Ichiro Hanamura, J. Luis Espinoza, Makoto Onizuka, Koichi Kashiwase, Yasuo Morishima, Takahiro Fukuda, Yoshihisa Kodera, Noriko Doki, Koichi Miyamura, Takehiko Mori, Seishi Ogawa, Akiyoshi Takami

**Affiliations:** ^1^ Division of Hematology, Department of Internal Medicine, Aichi Medical University School of Medicine, Nagakute, Japan; ^2^ Department of Pathology and Tumor Biology, Graduate School of Medicine, Kyoto University, Kyoto, Japan; ^3^ Cellular Transplantation Biology, Kanazawa University Graduate School of Medical Science, Kanazawa, Japan; ^4^ Department of Hematology and Oncology, Tokai University School of Medicine, Isehara, Japan; ^5^ Japanese Red Cross Kanto-Koshinetsu Block Blood Center, Tokyo, Japan; ^6^ Division of Epidemiology and Prevention, Aichi Cancer Center Research Institute, Nagoya, Japan; ^7^ Hematopoietic Stem Cell Transplantation Unit, National Cancer Center Hospital, Tokyo, Japan; ^8^ Department of Promotion for Blood and Marrow Transplantation, Aichi Medical University, Nagakute, Japan; ^9^ Hematology Division, Tokyo Metropolitan Cancer and Infectious Diseases Center Komagome Hospital, Tokyo, Japan; ^10^ Department of Hematology, Japanese Red Cross Nagoya First Hospital, Nagoya, Japan; ^11^ Division of Hematology, Department of Medicine, Keio University School of Medicine, Tokyo, Japan

**Keywords:** toll-like receptor, unrelated donor, bone marrow transplantation, single nucleotide variation

## Abstract

The Toll-like receptor family mediates the innate immune system through recognizing the molecular patterns of microorganisms and self-components and leading the synthesis of the inflammatory mediators. We retrospectively examined whether or not genetic variations in *toll-like receptor 1* (rs5743551, -7202GQ>A), *toll-like receptor 2* (rs7656411, 22215G>T), and *toll-like receptor 4* (rs11536889, +3725G>C) affected transplant outcomes in a cohort of 365 patients who underwent unrelated HLA-matched bone marrow transplantation (for hematologic malignancies through the Japan Marrow Donor Program. Only donor *toll-like receptor 4* variation significantly improved the survival outcomes. A multivariate analysis showed that the donor *toll-like receptor 4* +3725G/G genotype was significantly associated with a better 5-year progression-free survival and a lower 5-year transplant-related mortality than other variations. Furthermore, the donor *toll-like receptor 4* +3725G/G genotype was associated with a significantly lower incidence of fatal infections than other variations. The validation study of 502 patients confirmed that the donor *toll-like receptor 4* +3725G/G genotype was associated with better survival outcomes. *Toll-like receptor4* genotyping in transplant donors may therefore be a useful tool for optimizing donor selection and evaluating pretransplantation risks.

## INTRODUCTION

Allogeneic hematopoietic stem cell transplantation (SCT) is a potentially curative treatment for hematologic malignancies [1]. Despite HLA matching and substantial improvements in supportive care, life-threatening complications, including severe infections, organ damage and graft-versus-host disease (GVHD), remain an enormous obstacle [2]. There is growing evidence that non-HLA genetic variation involved in the immune response also represents a significant determinant of outcomes after SCT [3–8].

The toll-like receptor (TLR) family, the most important pattern recognition receptor family, plays a central role in sensing invading pathogens and tissue damage as danger signals, which leads to the massive release of inflammatory mediators such as pro-inflammatory cytokines, reactive oxygen species, antimicrobial peptides and acute-phase proteins into the bloodstream [9, 10]. TLRs are ubiquitously expressed on various cells, such as macrophages, dendritic cells, B cells, T cells, fibroblasts and epithelial cells. Previous reports [11–13] have suggested that TLRs contribute to the inflammatory processes after SCT, where subsequent translocation of bacterial components as well as release of endogenous danger molecules stimulate TLRs to trigger cytokine storm, leading to organ damage, which may vitiate the beneficial anti-microbial effect of TLRs.

Several *TLR* genes have been investigated in terms of the impact of their variation on outcome and susceptibility to infection and cancer. Among them, 3 functional variations [14–16] in the *TLR1* (rs5743551, -7202A>G), *TLR2* (rs7656411, 22215G>T) and *TLR4* (rs11536889, +3725G>C) genes with high (>0.2) minor allele frequencies in the Asian population were retrospectively investigated to determine their associations with transplant outcomes in a cohort of patients who underwent unrelated HLA-matched bone marrow transplantation (BMT) for hematologic malignancies through the Japan Marrow Donor Program (JMDP).

## RESULTS

### Frequencies of TLR genotypes

The rs5743551 (−7202G>A) variation in the *TLR1* gene, the rs7656411 (22215G>T) variation in the *TLR2* gene and the rs11536889 (+3725G>C) variation in the *TLR4* gene were genotyped in 365 recipients with hematologic malignancies and their unrelated donors in the discovery cohort (Table 1). The frequencies of A/A, A/G and G/G in the *TLR1* -7202G>A genetic variant were 13%, 44% and 44% in the recipients and 8.8%, 41% and 50% in the donors (*P*=0.52), respectively. The frequencies of G/G, G/T and T/T in the *TLR2* 22215G>T genetic variant were 28%, 52% and 19% in the recipients and 36%, 44% and 20% in the donors (*P*=0.43), respectively. The frequencies of C/C, C/G and G/G in the *TLR4* +3725G>C genetic variant were 7.4%, 41% and 52% in the recipients and 5.8%, 39% and 55% in the donors (*P*=0.85), respectively.

**Table 1:** Recipient and donor characteristics in the discovery and validation cohorts

Variable	Discovery cohort (n=365)	Validation cohort (n=502)	*P*
Number of cases	365	502	
Patient age, years, median (range)	36 (1-70)	35 (1-67)	
Donor age, years, median (range)	33 (20-51)		
Year of HSCT, median (range)	2002 (1993-2005)	2002 (1993-2005)	
Patient *TLR1* genotype, n (%)			
A/A	46 (13)		
A/G	160 (44)		
G/G	159 (44)		
Donor *TLR1* genotype, n (%)			
A/A	32 (8.8)		
A/G	150 (41)		
G/G	183 (50)		
Patient *TLR2* genotype, n (%)			
G/G	104 (28)		
G/T	191 (52)		
T/T	70 (19)		
Donor *TLR2* genotype, n (%)			
G/G	132 (36)		
G/T	161 (44)		
T/T	72 (20)		
Patient *TLR4* genotype, n (%)			0.26
C/C	27 (7.4)	15 (3.0)	
C/G	149 (41)	143 (28)	
G/G	189 (52)	344 (69)	
Donor *TLR4* genotype, n (%)			0.24
C/C	21 (5.8)	28 (5.6)	
C/G	142 (39)	135 (27)	
G/G	202 (55)	339 (68)	
Patient sex, n (%)			0.65
Male	225 (62)	317 (63)	
Female	140 (38)	185 (37)	
Donor sex, n (%)			0.53
Male	241 (66)	321 (64)	
Female	124 (34)	181 (36)	
Patient/Donor sex match, n (%)			0.43
Sex-matched	253 (69)	334 (67)	
Female/Male	64 (18)	86 (17)	
Male/Female	48 (13)	82 (16)	
Disease, n (%)			0.0021
AML	125 (34)	147 (29)	
ALL	82 (22)	154 (31)	
MDS	57 (16)	58 (12)	
ML	36 (9.9)	61 (12)	
CML	65 (18)	82 (16)	
Myeloid malignancies	247 (68)	287 (57)	
Lymphoid malignancies	118 (32)	215 (43)	
Disease stage, n (%)			
High risk	135 (37)		
Standard risk	230 (63)		
ABO matching, n (%)			
ABO-matched	238 (65)		
Major mismatch	61 (17)		
Minor mismatch	57 (16)		
Bidirectional	6 (1.6)		
Conditioning regimen, n (%)			0.015
Myeloablative	322 (88)	467 (93)	
Reduced intensity	43 (12)	35 (6.9)	
Pretransplantation CMV serostatus, n (%)			
CMV-positive recipient	224 (61)		
Missing	90 (25)		
TNC, ×10^8^/kg, median (range)	3.0 (0.08-12.3)		
Causes of fatal infections, n	10		
Pneumonia, unidentifiable	4		
Pneumonia, cytomegalovirus	2		
Pneumocystis pneumonia	1		
Brain abscess, fungal	1		
Sepsis, bacterial	2		

TNC, total number of nucleated cells harvested.

### Transplant outcomes according to TLR genotypes

**Figure 1:**
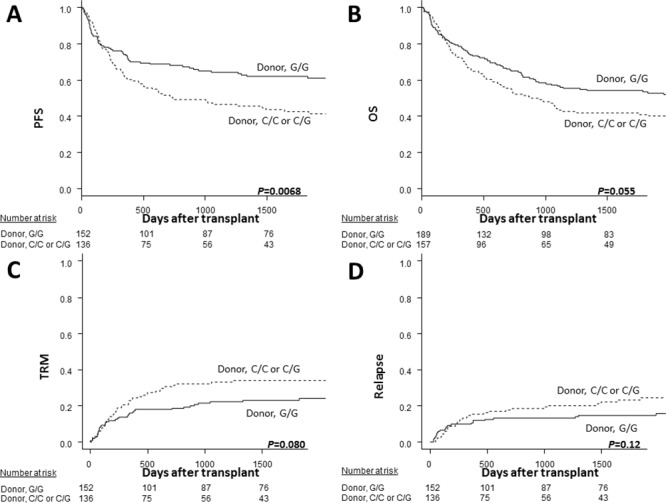
The Kaplan-Meier analysis of the progression-free survival rates **(A)** and the overall survival rates **(B)**, and the estimated incidence curves of transplant-related mortality **(C)** and disease relapse **(D)** after transplantation according to the donor *TLR4* genotype in the discovery cohort. The solid lines represent the donor G/G genotype, and the dashed lines represent the donor C/C or C/G genotype.

Univariate analyses (Tables 2 to 4) showed that only the donor *TLR4* genotype significantly improved the survival outcomes, with the donor *TLR4* +3725G/G genotype associated with a better 5-year PFS than the C/C or C/G genotype (62% *vs*. 43%, *P*=0.0068; Figure 1A). Five years was set as the study timepoint according to the median follow-up period among the survivors (970 days; range, 125 to 4798 days). The donor *TLR4* +3725G/G genotype also exhibited a trend toward a better 5-year OS (54% *vs*. 41%, *P*=0.055; Figure 1B) and a lower 5-year TRM (23% *vs*. 34%, *P*=0.080; Figure 1C) but did not reduce the 5-year relapse rate (15% *vs*. 23%, *P*=0.12; Figure 1D). The decrease in the number of analyzed cases regarding the transplant outcomes was due to the lack of data on the survival time and date of relapse in some cases. An analysis by mean imputation for missing data also showed that the donor *TLR4* +3725G/G genotype was associated with a better 5-year PFS (52% vs. 39%, *P*=0.034) and a trend toward a better 5-year OS (56% vs. 43%, *P*=0.051) compared to other genotypes (Supplementary Table 1), indicating that the presence of the missing data did not markedly impair the results. After adjusting for clinical factors in the multivariate model (Table 5), the donor *TLR4* +3725G/G genotype remained associated with a better 5-year PFS (HR, 0.57; 95% CI, 0.40-0.82; *P*=0.0022) and also showed tendencies toward a better 5-year OS (HR, 0.75; 95% CI, 0.55-1.0; *P*=0.061) and 5-year TRM (HR, 0.63; 95% CI, 0.40-1.0; *P*=0.057) than other variations. No other genotypes of *TLR1*, *TLR2* or *TLR4* significantly influenced the survival outcomes in multivariate analyses.

**Table 2:** The results of a univariate analysis regarding the association between *TLR1* variations and clinical outcomes after transplantation in the discovery cohort

Variable	n	5-year OS	*P*	5-year PFS	*P*	5-year TRM	*P*	5-year Relapse	*P*
**Recipient *TLR1* genotype**									
**A/A**	46	47%		57%		26%		17%	
**A/G**	160	47%	1.0	53%	1.0	25%	1.0	22%	1.0
**G/G**	159	49%	1.0	52%	1.0	32%	1.0	16%	1.0
**G/G**	159	49%		52%		32%		16%	
**A/G or A/A**	206	47%	0.79	54%	0.94	26%	0.41	20%	0.35
**Donor *TLR1* genotype**									
**A/A**	32	54%		62%		29%		8.7%	
**A/G**	150	47%	0.92	51%	0.67	28%	1.0	21%	0.70
**G/G**	183	47%	0.92	53%	0.67	28%	1.0	19%	0.70
**G/G**	183	47%		53%		28%		19%	
**A/G or A/A**	182	48%	0.81	53%	0.67	28%	0.83	19%	0.76

Variable	n	Grade II-IV acute GVHD	*P*	Grade III-IV acute GVHD	*P*	Chronic GVHD	*P*
**Recipient *TLR1* genotype**							
**A/A**	46	34%		9.1%		51%	
**A/G**	160	31%	1.0	11%	0.84	46%	1.0
**G/G**	159	38%	1.0	15%	0.84	46%	1.0
**G/G**	159	38%		15%		46%	
**A/G or A/A**	206	31%	0.25	11%	0.20	47%	0.98
**Donor *TLR1* genotype**							
**A/A**	32	18%		0.0%		56%	
**A/G**	150	39%	0.14	16%	0.080	50%	0.59
**G/G**	183	32%	0.29	12%	0.10	41%	0.41
**G/G**	183	32%		12%		41%	
**A/G or A/A**	182	36%	0.53	13%	0.85	51%	0.085

**Table 3:** The results of a univariate analysis regarding the association between *TLR2* variations and clinical outcomes after transplantation in the discovery cohort

Variable	n	5-year OS	*P*	5-year PFS	*P*	5-year TRM	*P*	5-year Relapse	*P*
**Recipient *TLR2* genotype**									
**G/G**	104	52%		58%		27%		16%	
**G/T**	191	47%	1.0	53%	1.0	30%	1.0	17%	0.96
**T/T**	70	46%	1.0	46%	1.0	25%	1.0	29%	0.62
**G/G**	104	52%		58%		27%		16%	
**G/T or T/T**	261	46%	0.93	51%	0.89	29%	0.82	20%	0.64
**Donor *TLR2* genotype**									
**G/G**	132	46%		53%		29%		19%	
**G/T**	161	52%	1.0	56%	1.0	27%	1.0	17%	1.0
**T/T**	72	42%	1.0	45%	1.0	31%	1.0	24%	1.0
**G/G**	132	46%		53%		29%		19%	
**G/T or T/T**	233	49%	0.91	53%	0.77	28%	0.90	19%	0.74

Variable	n	Grade II-IV acute GVHD	*P*	Grade III-IV acute GVHD	*P*	Chronic GVHD	*P*
**Recipient *TLR2* genotype**							
**G/G**	104	27%		8.2%		44%	
**G/T**	191	39%	0.13	14%	0.37	50%	0.47
**T/T**	70	32%	0.67	17%	0.29	39%	0.65
**G/G**	104	27%		8.2%		44%	
**G/T or T/T**	261	37%	0.070	14%	0.12	47%	0.42
**Donor *TLR2* genotype**							
**G/G**	132	25%		10%		47%	
**G/T**	161	39%	0.054	13%	1.0	51%	0.54
**T/T**	72	39%	0.166	16%	0.88	36%	0.48
**G/G**	132	25%		10%		47%	
**G/T or T/T**	233	39%	**0.015**	14%	0.36	46%	0.97

Underlined and bold results represent P <0.05.

Table 4The results of a univariate analysis regarding the association between *TLR4* variations and clinical outcomes after transplantation in the discovery cohort

**Table:** 

Variable	n	5-year OS	*P*	5-year PFS	*P*	5-year TRM	*P*	5-year Relapse	*P*
**Recipient *TLR4* genotype**									
**G/G**	189	48%		52%		30%		18%	
**C/G**	149	50%	0.66	57%	0.49	25%	0.83	18%	1.0
**C/C**	27	39%	0.33	43%	0.49	33%	0.83	24%	1.0
**G/G**	189	48%		52%		30%		18%	
**C/G or C/C**	176	48%	0.98	54%	0.68	26%	38	19%	0.65
**Donor *TLR4* genotype**									
**G/G**	202	54%		62%		23%		15%	
**C/G**	142	41%	0.23	43%	0.035	35%	0.25	23%	0.50
**C/C**	21	42%	0.72	44%	0.17	28%	0.85	29%	0.50
**G/G**	202	54%		62%		23%		15%	
**C/G or C/C**	163	41%	0.055	43%	**0.0068**	34%	0.080	23%	0.12

Underlined and bold results represent *P* <0.05.

**Table:** 

Variable	n	Grade II-IV acute GVHD	*P*	Grade III-IV acute GVHD	*P*	Chronic GVHD	*P*
**Recipient *TLR4* genotype**							
**G/G**	189	32%		14%		49%	
**C/G**	149	38%	0.60	12%	1.0	47%	1.0
**C/C**	27	26%	0.60	7.4%	1.0	24%	0.14
**G/G**	189	32%		14%		49%	
**C/G or C/C**	176	36%	0.34	12%	0.58	43%	0.53
**Donor *TLR4* genotype**							
**G/G**	202	32%		11%		44%	
**C/G**	142	38%	0.68	15%	0.63	49%	1.0
**C/C**	21	33%	1.0	19%	0.63	53%	1.0
**G/G**	202	32%		11%		44%	
**C/G or C/C**	163	37%	0.24	15%	0.18	50%	0.47

Table 5The results of a multivariate analysis regarding the association between *TLR1*, *TLR2* and *TLR4* variations and clinical outcomes after transplantation in the discovery cohort

**Table:** 

Variable	5-year OS			5-year PFS			5-year TRM			5-year Relapse		
	HR	95% CI	*P*	HR	95% CI	*P*	HR	95% CI	*P*	HR	95% CI	*P*
**Recipient *TLR1* genotype, G/G *vs*. A/A or A/G**	1.00	0.74-1.40	0.96	1.00	0.70-1.40	0.97	1.29	0.80-2.00	0.31	0.63	0.31-1.30	0.21
**Donor *TLR1* genotype,G/G vs. A/A or A/G**	1.09	0.80-1.40	0.59	1.16	0.81-1.60	0.42	1.22	0.78-1.92	0.38	1.06	0.55-2.00	0.87
**Recipient *TLR2* genotype, G/G *vs*. T/T or G/T**	1.02	0.59-1.80	0.95	1.04	0.71-1.50	0.86	1.34	0.83-2.10	0.23	0.61	0.24-1.50	0.28
**Donor *TLR2* genotype,G/G *vs*. T/T or G/T**	1.24	0.71-2.10	0.45	1.24	0.85-1.80	0.28	1.17	0.71-1.90	0.54	0.84	0.40-1.80	0.65
**Recipient *TLR4* genotype, G/G *vs***. **C/C or C/G**	1.00	0.74-1.30	0.98	1.06	0.75-1.50	0.74	1.09	0.68-1.73	0.72	0.65	0.34-1.30	0.20
**Donor *TLR4* genotype,G/G *vs***. **C/C or C/G**	0.75	0.55-1.00	0.061	0.57	0.40-0.82	**0.0022**	0.63	0.40-1.00	0.057	0.62	0.31-1.20	0.17
Recipient age	1.03	1.00-1.00	<0.001	1.03	1.00-1.00	<0.001	1.04	1.00-1.10	<0.001	1.01	0.98-1.05	0.38
Year of HSCT	0.78	0.55-1.10	0.17				0.52	0.31-0.89	0.016			
Recipient/Donor sex match												
Female/Male										0.36	0.13-1.00	0.053
Male/Female										0.13	0.018-0.95	0.044
Disease stageStandard risk/High risk	2.01	1.50-2.70	<0.001	1.90	1.30-2.70	<0.001	2.06	1.30-3.30	0.0021			
ABO Major mismatch							0.69	0.34-1.40	0.29			
ABO Minor mismatch							1.80	0.98-3.30	0.058			
ABO Bidirectional							<0.001	0.00-0.00	0.00			
Conditioning regimen MAC/RIC							0.64	0.30-1.4	0.24			
Pretransplantation CMV serostatus												
CMV-positive recipient										1.70	0.54-5.3	0.36
Missing										24.15	7.0-84	<0.001
TNC	1.00	1.0-1.0	0.38	1.00	1.0-1.0	0.90	1.00	1.0-1.0	0.33			

TNC, total number of nucleated cells harvested.

Underlined and bold results regarding the genotype represent *P* <0.05.

**Table:** 

Variable	Grades II-IV acute GVHD			Grades III-IV acute GVHD			Chronic GVHD		
	HR	95% CI	*P*	HR	95% CI	*P*	HR	95% CI	*P*
**Recipient *TLR1* genotype,G/G *vs*. A/A or A/G**	1.21	0.83-1.80	0.33	1.57	0.87-2.80	0.13	1.05	0.75-1.50	0.77
**Donor *TLR1* genotype,G/G vs. A/A or A/G**	0.89	0.61-1.30	0.55	0.93	0.51-1.70	0.80	0.76	0.53-1.10	0.12
**Recipient *TLR2* genotype,G/G *vs*. T/T or G/T**	0.67	0.43-1.10	0.084	0.53	0.25-1.10	0.097	0.82	0.56-1.20	0.30
**Donor *TLR2* genotype,G/G *vs*. T/T or G/T**	0.62	0.41-0.95	**0.028**	0.70	0.36-1.40	0.31	0.97	0.68-1.40	0.87
**Recipient *TLR4* genotype,G/G *vs***. **C/C or C/G**	0.86	0.59-1.30	0.43	1.21	0.65-2.20	0.55	1.07	1.00-2.20	0.043
**Donor *TLR4* genotype,G/G *vs***. **C/C or C/G**	0.84	0.58-1.20	0.35	0.67	0.37-1.20	0.18	0.87	0.62-1.20	0.43
Recipient age	0.99	0.98-1.00	0.11						
Donor age	1.03	1.00-1.10	0.022						
Disease stageStandard risk/High risk	1.22	0.84-1.80	0.31	1.54	0.85-2.80	0.16			
Myeloid malignancies							1.50	1.00-2.20	0.043
TNC	1.00	1.00-1.00	0.15						

TNC, total number of nucleated cells harvested.

Underlined and bold results regarding the genotype represent *P* <0.05.

**Figure 2:**
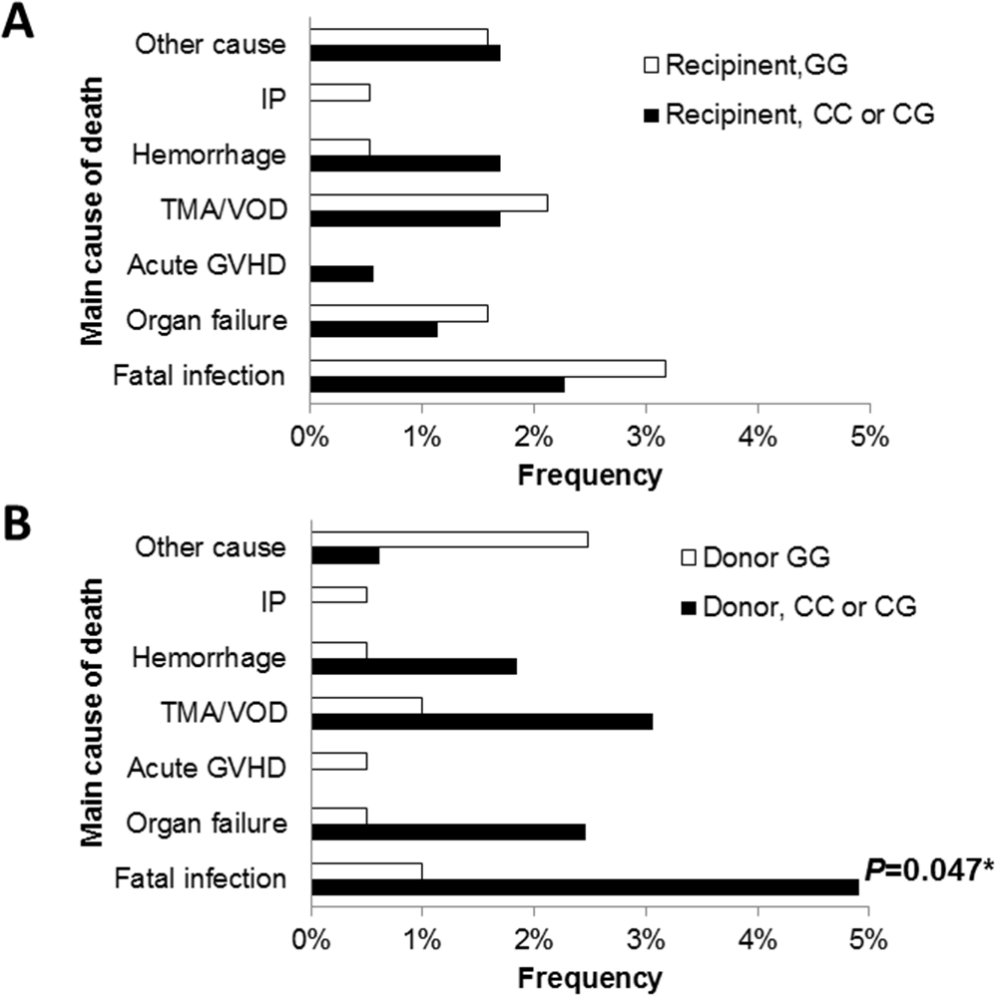
The main causes of death after transplantation according to the recipient **(A)** and donor *TLR4* genotype **(B)** in the discovery cohort. The asterisk denotes *P* <0.05.

**Figure 3:**
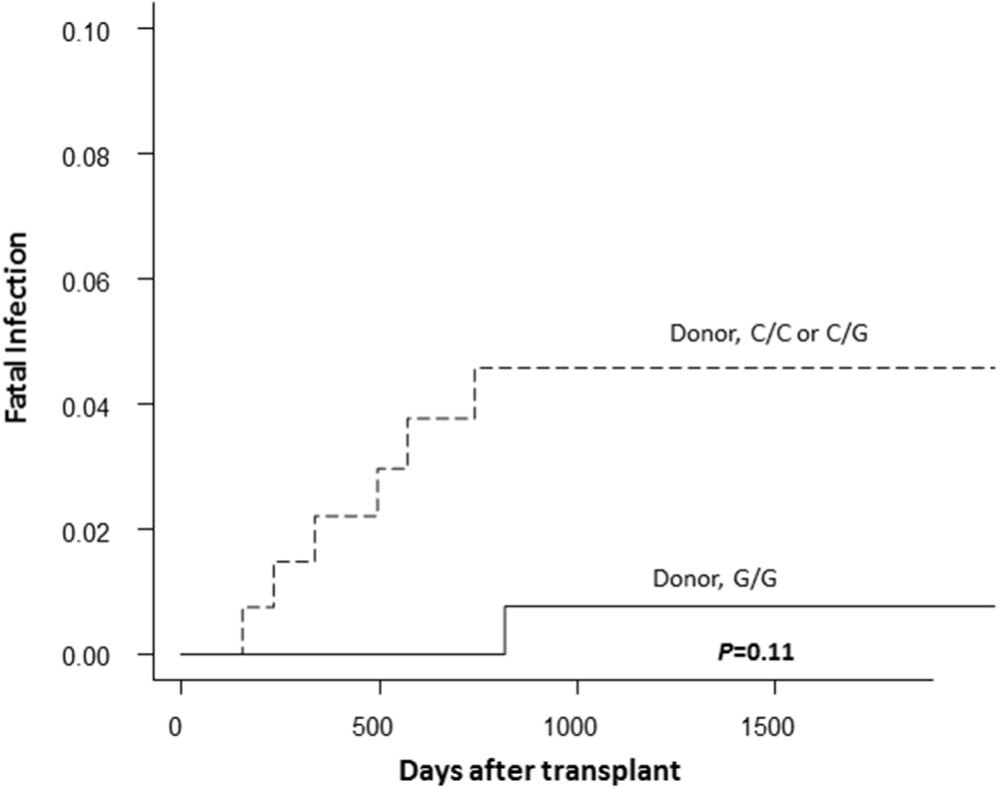
The cumulative incidence of fatal infection after transplantation according to the donor TLR4 genotype in the discovery cohort The solid lines represent the donor G/G genotype, and the dashed lines represent the donor C/C or C/G genotype.

When the main causes of TRM were analyzed according to the *TLR4* +3725G>C genotype, the donor *TLR4* +3725G/G genotype was associated with a significantly lower incidence of fatal infections than other genotypes (*P*=0.047; Figure 2). The donor *TLR4* +3725G/G genotype resulted in one fifth of the cumulative incidence of fatal infection (0.7% *vs*. 4.6%; *P*=0.11; Figure 3), although there were no statistical differences.

### The validation cohort study

**Figure 4:**
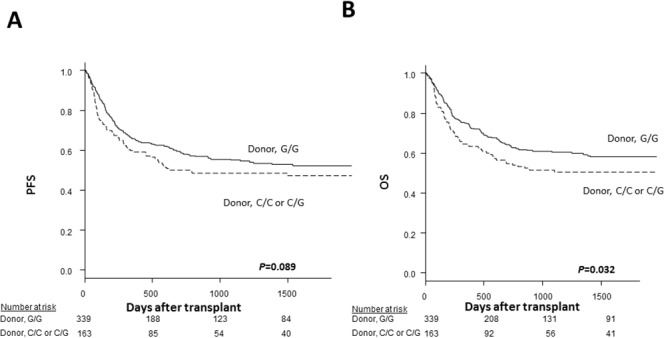
The Kaplan-Meier analysis of the progression-free survival rates **(A)** and the overall survival rates **(B)** after transplantation according to the donor *TLR4* genotype in the validation cohort. The solid lines represent the donor G/G genotype, and the dashed lines represent the donor C/C or C/G genotype.

The characteristics of the patients in the validation cohort were similar to those of the patients in the discovery cohort with the exception that the donors in the validation cohort were HLA-DPB1 allele-mismatched. The donor *TLR4* +3725G/G genotype was associated with a better 5-year OS (58% *vs*. 51%, *P*=0.032; Figure 4B; Table 6) as well as a trend toward better 5-year PFS (52% *vs*. 47%, *P*=0.089; Figure 4A). According to a multivariate analysis, the donor *TLR4* +3725G/G genotype remained associated with a significantly better 5-year OS (HR, 0.75; 95% CI, 0.56-0.99; *P*=0.043; Table 7) and moderately better 5-year PFS (HR, 0.81; 95% CI, 0.62-1.1; *P*=0.12).

Table 6The results of a univariate analysis regarding the association between *TLR4* variations and clinical outcomes after transplantation in the validation cohort

**Table:** 

Variable	n	5-Year OS, %	*P*	5-Year PFS, %	*P*	5-Year TRM, %	*P*	5-Year Relapse, %	*P*
**Recipient *TLR4* genotype**									
**G/G**	344	55		50		28		21	
**C/G**	143	56	1.0	50	1.0	29	1.0	21	1.0
**C/C**	15	60	1.0	60	1.0	20	1.0	20	1.0
**G/G**	344	55		50		28		21	
**C/G or C/C**	158	56	0.94	51	0.78	28	0.94	21	0.90
**Donor *TLR4* genotype**									
**G/G**	339	58		52		27		21	
**C/G**	135	51	0.13	48	0.39	30	0.72	22	1.0
**C/C**	28	50	0.54	42	0.57	39	0.64	20	1.0
**G/G**	339	58		52		27		21	
**C/G or C/C**	163	51	**0.032**	47	0.089	31	0.22	22	0.58

Underlined and bold results represent *P* <0.05.

**Table:** 

Variable	n	Grade II-IV aGVHD, %	*P*	Chronic GVHD, %	*P*
**Recipient *TLR4* genotype**					
**G/G**	344	49		42	
**C/G**	143	50	1.0	38	0.84
**C/C**	15	40	1.0	27	0.84
**G/G**	344	49		42	
**C/G or C/C**	158	49	0.59	37	0.38
**Donor *TLR4* genotype**					
**G/G**	339	49		38	
**C/G**	135	49	1.0	50	0.13
**C/C**	28	50	1.0	25	0.13
**G/G**	339	49		38	
**C/G or C/C**	163	49	0.95	45	0.31

Table 7The results of a multivariate analysis regarding the association between *TLR4* variations and the clinical outcomes after transplantation in the validation cohort

**Table:** 

Variable	5-year OS			5-year PFS			5-year TRM			5-year Relapse		
	Adjusted HR	95%CI	*P*	Adjusted HR	95%CI	*P*	Adjusted HR	95%CI	*P*	Adjusted HR	95%CI	*P*
Recipient *TLR4* genotype, G/G *vs*. C/C or C/G	1.0	0.75-1.3	1.0	0.97	0.74-1.3	0.83	0.98	0.68-1.4	0.90	0.98	0.64-1.5	0.93
Donor *TLR4* genotype, G/G *vs*. C/C or C/G	0.75	0.56-0.99	**0.043**	0.81	0.62-1.1	0.12	0.81	0.57-1.1	0.24	0.87	0.56-1.3	0.53
Recipient age	1.4	1.0-1.8	0.026	1.3	1.0-1.7	0.033	1.9	1.3-2.7	0.00038	0.75	0.50-1.1	0.17
Recipient/Donor sex match	1.1	0.91-1.3	0.38	1.1	0.92-1.3	0.3						
Female/Male							0.85	0.53-1.4	0.49	1.1	0.67-1.9	0.61
Male/Female							1.1	0.72-1.8	0.57	1.3	0.75-2.1	0.39

Underlined and bold results regarding the genotype represent *P* <0.05.

**Table:** 

Variable	Grades II-IV acute GVHD			Grades III-IV acute GVHD			Chronic GVHD		
	Adjusted HR	95%CI	*P*	Adjusted HR	95%CI	*P*	Adjusted HR	95%CI	*P*
Recipient *TLR4* genotype, G/G *vs*. C/C or C/G	0.93	0.70-1.2	0.61	0.94	0.58-1.5	0.79	1.2	0.84-1.6	0.35
Donor *TLR4* genotype, G/G *vs*. C/C or C/G	1.0	0.78-1.3	0.89	0.78	0.49-1.2	0.29	0.85	0.62-1.2	0.29
Recipient age	1.0	0.82-1.4	0.64	1.1	0.68-1.7	0.76	1.2	0.92-1.7	0.15
Recipient/Donor sex match									
Female/Male	1.1	0.82-1.6	0.46	1.1	0.62-2.1	0.69	1.1	0.73-1.6	0.70
Male/Female	0.9	0.63-1.3	0.57	1.1	0.59-2.0	0.81	0.96	0.62-1.5	0.85

## DISCUSSION

The discovery cohort study, which consisted of recipient and donor pairs for which the HLA-A, -B, -C, -DRB1, -DQB1, and -DPB1 alleles were completely matched, revealed that the donor G/G genotype at rs11536889 (+3725G>C) of the *TLR4* gene predicts significantly better 5-year PFS than other genotypes in patients with hematological malignancies receiving unrelated BMT. The beneficial effects of the donor *TLR4* +3725G/G genotype were seen on TRM and death attributable to infections but were not evident with respect to GVHD, suggesting that the donor *TLR4* +3725G/G genotype helps prevent fatal infections. The validation cohort study, which consisted of recipient and donor pairs with one or two HLA-DPB1 mismatched alleles, showed consistent results with the donor *TLR4* +3725G/G genotype associated with better survival outcomes.

The mechanisms through which the donor *TLR4* +3725G/G genotype exerts its beneficial effects remain to be determined. A recent report [16] demonstrated that the *TLR4* +3725G>C variation located in its 3′-untranslated region was functional, and monocytes from *TLR4* +3725G/G subjects expressed lower levels of TLR4 on their surfaces and were less responsive to lipopolysaccharide (LPS), a TLR4 ligand, than those from C/G or C/C subjects, possibly due to the preference of microRNAs for binding to the +3725G allele over the +3725C allele. The lower translational activity associated with the *TLR4* +3725G/G genotype may contribute to prevent infection-associated death following SCT.

Evidence of TLR4 activation having a negative influence on outcomes following severe infection has been demonstrated in previous studies using mouse models [17–19], showing that blockade of TLR4 inhibited systemic inflammatory responses in sepsis, which reduced the disease severity and lethality. Although one may deduce from these findings that the putatively hypoactive *TLR4* +3725G/G genotype increases susceptibility to infectious diseases, the *TLR* +3725G/G genotype was associated with a lower risk of developing sepsis in China [20], suggesting this to be unlikely. This hypothesis may also be supported by the association of the *TLR* +3725G/G genotype with a lower rate of Gram-negative infection in sepsis in Germany [21], and with a lower susceptibility to severe gastric atrophy related to *Helicobacter pylori* infection [22, 23], hepatitis B recurrence after liver transplantation [24] and advanced periodontitis [25, 26] in the Asian populations. Of note, the *TLR* +3725G/G genotype was beneficially associated with a lower risk of prostate cancer in Sweden [27] and a lower risk of developing chemotherapy-induced neutropenia in children with ALL in Netherland [28], although their etiological relevance to the current findings are unclear. In the present study, the *TLR* +3725G>C variation did not significantly influence engraftment and relapse rates after SCT.

Bochud, et al.[29] reported that donor *TLR4* haplotype S4 among haplotypes S1 to S4 was associated with a risk of invasive aspergillosis after allogeneic hematopoietic stem cell transplantation. However, because haplotype S4 is absent in Asian populations [30, 31], which is defined by the *TLR4* variations rs4986790 [D299G] and rs4986791 [T399I], but not by *TLR4* variation rs11536889 that was investigated in the present study, we decided not to investigate the potential link between each haplotype and the transplant outcomes.

One major limitation associated with the present study is that the detailed information on the infections, including the types, severity, treatments, and therapeutic appropriateness, was beyond the scope of the current study and the only the information on the causes of death (such as fatal infections) was available.

In conclusion, the findings of the present data suggested that the donor *TLR4* +3725G>C variations predicted better survival outcomes after SCT than other genotypes. Therefore, *TLR4* +3725G>C genotyping in donors may be a valuable tool for selecting donors and evaluating pretransplantation risks that, combined with other currently known risk factors, can form the basis for carrying out suitable tailoring of transplantation strategies. Considering the plausible functional roles of these variations, they may be candidates for future prophylactic and therapeutic strategies for complications after allogeneic SCT. Further studies are warranted to ascertain whether or not the findings of this study can be extended to other stem cell sources or to HLA-mismatched transplantation and to validate the present findings in other ethnic groups.

## PATIENTS AND METHODS

### Patients

The patients underwent BMT through the JMDP with T cell-replete marrow between May 1993 and November 2005 (Table 1). No patients had a history of any prior transplantation. The final clinical data analyses of these patients were completed by November 25, 2009. The conditioning regimen varied according to the underlying disease and the condition of the patient. The combination of cyclophosphamide (CY) combined with total body irradiation (TBI) was mainly used for the myeloablative conditioning (MAC) regimen, whereas the combination of fludarabine and melphalan or busulfan was mainly used for the reduced-intensity conditioning (RIC) regimen [32]. Cyclosporine or tacrolimus with short-term methotrexate was used for GVHD prophylaxis [33, 34]. No patients received anti-T cell therapy, such as antithymocyte globulin or *ex vivo* T cell depletion, in this study. All patients and donors gave their informed consent at the time of transplantation to take part in molecular studies of this nature, in accordance with the Declaration of Helsinki. This project was approved by the Institutional Review Board of Aichi Medical University School of Medicine and the JMDP. All methods were performed in accordance with the approved guidelines and regulations.

For the discovery study, *TLR1*, *TLR2* and *TLR4* genotyping was performed in 365 patients with hematologic malignancies and their unrelated and HLA-A, HLA-B, HLA-C, HLA-DRB1, HLA-DQB1 and HLA-DPB1 allele-matched donors. The diagnoses included acute myeloid leukemia (AML) (n=125; 34%), acute lymphoblastic leukemia (ALL) (n=82; 22%), myelodysplastic syndrome (MDS) (n=57; 16%), malignant lymphoma (ML) (n=36; 9.9%) and chronic myeloid leukemia (CML) (n=65; 18%). “Standard risk” included acute leukemia in the first remission, chronic myeloid leukemia in the chronic phase and myelodysplastic syndrome and malignant lymphoma in complete remission. “High risk” included all others. Lymphoid malignancies included ALL and ML.

For the validation study, the *TRL4* rs11536889 variations were imputed using the data from the 1000 Genomes Project 36, as described in our previous study [35]. The cohort included 502 patients with hematological malignancies and their unrelated donors who were HLA-A, HLA-B, HLA-C, HLA-DRB1, and HLA-DQB1 allele-matched but who were mismatched by one or two HLA-DPB1 alleles. The diagnoses included AML (n=149; 30%), ALL (n=152; 30%), MDS (n=58; 12%), ML (n=61; 12%) and CML (n=82; 16%).

HLA 12/12-allele-matched transplants were incorporated into the discovery cohort to eliminate the impact of HLA-allele mismatch on transplant outcomes, and all 365 available pairs were analyzed in the discovery cohort study. In conducting the validation study, in order to minimize the influence of HLA on the transplant outcomes, all 502 transplants from HLA-DPB1-mismatched donors were incorporated into the validation cohort.

### Genotyping

Real-time polymerase chain reaction (PCR) genotyping for *TLR1*, *TLR2* and *TLR4* was performed using the TaqMan-Allelic discrimination method in the StepOnePlus Real-Time PCR system (Applied Biosystems, Foster City, CA, USA) as described previously [6], and the results were analyzed using the Allelic Discrimination software program (Applied Biosystems). The specific probe designed for SNP rs5743551 (−7202G>A) (product No. C__1180670_30), rs7656411 (22215G>T) (product No. C__29420880_10), rs11536889 (+3725G>C) (product No. C__31784034_10) and TaqMan genotyping master mix were purchased from Applied Biosystems.

### Data management and statistical analyses

Data were collected by the JMDP using a standardized report form. Follow-up reports were submitted at 100 days, 1 year and then annually after transplantation. Only recipients were routinely measured for the pretransplantation cytomegalovirus (CMV) serostatus. The time to neutrophil engraftment was defined as the first of 3 consecutive days with an absolute neutrophil count of more than 0.5×10^9^/L. Acute GVHD developing within the first 100 days posttransplantation was diagnosed and graded based on the established criteria [36]. A classification of chronic GVHD observed in patients who survived beyond day 100 was based on the Seattle criteria [37]. The overall survival (OS) was calculated from the date of transplantation to the date of death from any cause. Disease relapse was defined as the number of days from transplantation to disease relapse or progression. The transplant-related mortality (TRM) was defined as death due to any cause other than relapse or disease progression. The PFS was defined as survival without disease relapse or progression. Any patients who were alive at the last follow-up date were censored. Data regarding the clinical and microbiological characteristics of infections, postmortem changes, prophylaxis against infections and therapy for GVHD given on an institutional basis were not considered in this study.

All of the statistical analyses were carried out using the EZR software package [38]. The probabilities of OS and PFS were calculated using the Kaplan-Meier method, and comparisons between groups were performed via the log-rank test. The occurrence of TRM, disease relapse, acute GVHD and chronic GVHD were compared using the Gray test [39] and analyzed using the cumulative incidence analysis [40], considering relapse, death without disease relapse, death without acute GVHD, death without chronic GVHD and death without engraftment as respective competing risks. The cumulative incidence of fatal infection was analyzed using the Gray test, while considering fatal infections without disease relapse and relapse or death without fatal infection as competing risks. A multivariate Cox model was constructed for the OS and PFS, and a Fine-Gray competing risk regression model was constructed for TRM, relapse, grades 2-4 acute GVHD, grades 3-4 acute GVHD and chronic GVHD using stepwise selection at a significance level of 5% to evaluate the hazard ratio (HR) associated with the *TLR1*, *TLR2* and *TLR4* genotypes. Recipient age at the time of BMT, sex, pretransplantation CMV serostatus, disease characteristics (i.e. disease type, disease lineage and disease risk at transplantation), donor characteristics (i.e. age, sex compatibility and ABO compatibility), transplant characteristics (i.e. MAC or RIC and total number of nucleated cells harvested per recipient weight) and year of transplantation were used as covariates. Although these covariates were all available for the discovery study, some of these covariates were not available for the validation study (Table 1). The median was used as the cut-off point for continuous variables. The chi-squared and Mann-Whitney tests were used to compare the results of two groups. The linkage disequilibrium (LD) structure among the single nucleotide polymorphisms in the *TLR1*, *TLR2* and *TLR4* gene was determined using HAPLOVIEW, version 4.2 [41, 42]. For all analyses, *P*<0.05 was considered statistically significant.

This study was supported by grants from the Ministry of Education, Culture, Sports and Technology of Japan (15K09513), the Ministry of Health, Labour and Welfare of Japan (15eK0510002h0002, 15ek0109134h0001, and 16mk0101065h0001), the SENSHIN Medical Research Foundation (Osaka, Japan), the Aichi Cancer Research Foundation (Nagoya, Japan) and the 24th General Assembly of the Japanese Association of Medical Sciences (Nagoya, Japan). The funders played no role in the study design, data collection and analysis, the decision to publish or the preparation of the manuscript.

## SUPPLEMENTARY MATERIALS TABLES


