# Supplementary material for: Toll-like receptor genetic variations in bone marrow transplantation
Source: Oncotarget. 2017 Apr 21;8(28):45670–86. doi: 10.18632/oncotarget.17315 (PMC5542217; doi:10.18632/oncotarget.17315)
Supplement: Supplementary file 1 [file oncotarget-08-45670-s001.pdf]

Toll-like receptor genetic variations in bone marrow transplantation

SUPPLEMENTARY TABLE

Supplementary Table 1: The results of a univariate analysis by using mean imputation for missing data regarding the association between *TLR4* variations and clinical outcomes after transplantation in the discovery cohort

Variable	n	5-year OS	*P*	5-year PFS	*P*	5-year TRM	*P*	5-year Relapse	*P*
**Recipient *TLR4* genotype**									
**G/G**	189	50%		45%		26%		30%	
**C/G**	149	53%	0.40	52%	0.26	23%	0.79	26%	1.0
**C/C**	27	37%	0.19	33%	0.081	33%	0.70	33%	0.97
**G/G**	189	50%		45%		26%		30%	
**C/G or C/C**	176	51%	0.79	49%	0.59	24%	0.65	27%	0.72
**Donor *TLR4* genotype**									
**G/G**	202	56%		52%		22%		26%	
**C/G**	142	44%	0.24	40%	0.19	30%	0.63	31%	0.71
**C/C**	21	42%	0.28	38%	0.47	24%	1.0	39%	0.71
**G/G**	202	56%		52%		22%		26%	
**C/G or C/C**	163	43%	0.051	39%	**0.034**	29%	0.20	32%	0.24

Underlined and bold results represent P <0.05.
